# Is Schistosomiasis a Risk Factor for Bladder Cancer? Evidence-Based Facts

**DOI:** 10.1155/2020/8270810

**Published:** 2020-06-01

**Authors:** Mohamed Jalloh, Ayun Cassell, Thierno Diallo, Omar Gaye, Medina Ndoye, Mouhamadou M. Mbodji, Mahamat Ali Mahamat, Abdourahmane Diallo, Cherif Dial, Issa Labou, Lamine Niang, Serigne M. Gueye

**Affiliations:** ^1^Service d'Urologie, Hopital General de Grand Yoff, Dakar, Senegal; ^2^Institut de Formation en Urologie et Santé Familiale, Dakar, Senegal; ^3^Centre Hospitalo-Universitaire de Référence Nationale, N'jamena, Chad

## Abstract

*Background* . Globally, approximately 20% of malignancy are caused by infection. Schistosoma infection is a major cause of bladder in most part of Africa. In 2018 alone, there were approximately 549,393 new cases and 199,922 deaths from bladder cancer. The presence of Schistosoma ova in the venous plexus of the bladder induces a cascade of inflammation causing significant tissue damage and granulomatous changes. *Methodology*. A literature review was conducted from 1995 to 2019 using PubMed, Google Scholar, African Journal Online, and Google databases. Relevant data on the association of “Schistosomiasis and Bladder cancer” in sub-Saharan Africa (SSA) were retrieved. *Evidence Synthesis*. Results from research using animal models to establish the carcinogenesis of Schistosoma and bladder cancer have been helpful but inconclusive. Immunoregulatory cytokines and genetic marker have been identified to play a role in the pathogenesis. In some parts of sub-Saharan Africa, there has been close association of squamous cell carcinoma and histological evidence of Schistosoma ova. *Conclusion*. There are some data to support the association between schistosomiasis and bladder cancer in sub-Saharan Africa. However, these have been limited by their design and may not sufficiently establish carcinogenesis. There is a need for more genomic and molecular research to better characterize *S. haematobium* and its effects on the bladder. Such goal will contribute immensely to Schistosoma bladder cancer prevention and control.

## 1. Background

Bladder cancer is an essential global health problem with an estimated 549,393 new cases and 199,922 deaths in 2018 [1]. The global age standardized rate was 9.6 per 100,000 for men and 2.4 per 100,000 for women in 2018 according to GLOBOCAN [1]. In 2019, a systemic review and meta-analysis by Adeloye et al. [2] revealed a pooled incidence of bladder cancer in Africa at 7.0 (95% confidence interval: 5.8–8.3/100,000 population) in males and 1.8 (95% confidence interval: 1.2–2.6/100,000) in females. Amongst Egyptian males, bladder cancer is the most common malignancy accounting for 16% with more than 7,900 deaths yearly [3]. The age standardized rate for Egyptian males with bladder cancer is about 27.9/100,000, which is the highest in Africa and the Middle East [3].

Globally, approximately 20% of malignancy are caused by infection. Schistosoma which is a blood fluke has an established endemicity in Africa and the Middle East, especially Egypt. In 1920, the rate of schistosomiasis in Egypt was as high as 80%, but it has drastically reduced to about 1.2% in 2006 due to the introduction of praziquantel through the National Schistosomiasis Control Program. Schistosomiasis was first discovered in 1851 by Theodor Bilharz. It was later identified as a cause of bladder cancer in 1911 by Fergusson [4]. In 1994, the International Agency for Research on Cancer (IARC) confirmed that *Schistosoma haematobium* (*S. haematobium*) was carcinogenic [4].

Most African nations have failed to reduce or terminate the transmission of schistosomiasis despite its close association with bladder cancer. The World Health Organization (WHO) in 2002 endorsed the Schistosomiasis Control Initiative (SCI) across several African states, but any tangible result is yet to be achieved [2]. These programs have failed in sub-Saharan Africa (SSA) due to insufficient awareness, poor coverage of chemoprophylaxis, and constant exposure to Schistosoma infection amongst children.

The risk and etiologies of bladder cancer vary considerably across sub-Saharan Africa from smoking and occupational exposures to schistosomiasis endemicity. A review by Bowa et al. in 2018 revealed that squamous cell carcinoma (SCC) in Africa is still strongly associated with schistosomiasis infection in up to 85% of cases [5]. Results from a contemporary review of bladder cancer in Africa showed that Schistosoma endemicity was a major etiological factor of bladder in nations such as Nigeria, Kenya, and Zambia [6]. In Mali, Zambia, Tanzania, and Nigeria, 10%–45% of patients diagnosed of squamous cell carcinoma of the bladder had associated Schistosoma cystitis [6]. The histological type in these regions is more SCC than urothelial cancer due to comparatively lower exposure to aromatic hydrocarbons and industrialized petrochemical in rural settings and higher exposure to Schistosoma infection.

This review assesses the presentation, etiopathogenesis, molecular basis, and histological evidence of schistosomiasis in bladder cancer in the sub-Saharan region.

## 2. Methodology

The literature review was conducted from 1995 to 2019 using the various search engines and academic databases. These included PubMed, Google Scholar, African Journal Online, and Google electronic database. The search strategy included the medical search heading (MEsH) “Schistosomiasis and Bladder cancer.” These keywords were appended sub-Saharan Africa, East Africa, West Africa, and Southern Africa to specify the search results. In addition, the references of each publication retrieved were scrutinized to look for other relevant studies. A total of 34 studies were selected for the review based on the presentation, etiopathogenesis, and histology of schistosomiasis and bladder cancer. These publications included systemic reviews, meta-analysis, review articles, and cohort and retrospective publications. Nine studies from the sub-Saharan region were selected and analyzed qualitatively for urinary schistosomiasis and bladder cancer. Each study methodology was reviewed and summarized. The results were analyzed to establish the relationship of schistosomiasis and bladder cancer in these regions. These findings are represented in Table 1 and evidence are synthesized in the main text of the discussion. The histopathological profile of bladder cancer and evidence of Schistosoma ova were the essential data retrieved from these 9 research papers.

The etiopathogenesis of Schistosoma-induced bladder cancer was elaborated from Asiatic and Western literature due to the paucity of data from sub-Saharan Africa (SSA). Schistosomiasis, carcinogenesis, and coinfection were also reviewed and analyzed from the published data.

Genetic or molecular studies related to Schistosoma and associated bladder cancer from the sub-Saharan region were not available from the searched literature to be analyzed. Immunohistochemical characteristics of Schistosoma bladder tumor were rather extrapolated from the studies published in Egypt and western nations.

## 3. Presentation

The clinical manifestation of SCC presents with mostly painful hematuria, bladder mass, and necroturia, while urothelial carcinoma of the bladder is mostly with painless hematuria [5]. Like most urological pathology in low-income countries, SCC presents late to the urologist when the disease is already advanced or metastatic. In addition, the pathogenesis of urothelial carcinoma suggests that the disease spread contiguously from the mucosa to the serosal layer or inward to outward [5]. In contrast, SCC occurs with the Schistosoma eggs implanted in the perivesical plexus, and the pathology seems to spread routinely in an opposite direction.

## 4. Etiopathogenesis of Schistosomiasis-Induced Bladder Cancer

The pathology of urogenital schistosomiasis primarily results from Schistosoma haematobium eggs laid by adult worms residing in the venous plexus of the bladder and other organs in the pelvis. The presence of these ova in the venous plexus induces a cascade of inflammation causing significant tissue damage and granulomatous changes. The prolonged inflammatory process leads to progressive tissue fibrosis, genetic changes, and subsequently bladder cancer [16].

Many studies have tried to establish the pathogenesis of Schistosoma haematobium and bladder cancer using animal models. Research by Fu et al. [17] showed some immunopathy (urothelial hyperplasia, tissue fibrosis, and dysfunction) after injection of Schistosoma egg to the mice model. However, results have shown that pelvic organs of animal models do not mount significant inflammatory reaction when injected with *Schistosoma haematobium* ova as compared to humans who have continuous oviposition and inflammatory response [16]. This places a limitation on these studies achieving any relevant results. To the contrary, *Clonorchis sinensis* and *Opisthorchis viverrini*-infected hamsters showed mounted inflammation and carcinogenesis when exposed to nitrosamine [16]. Appropriate level of inflammation is necessary to maintain adequate immunity to an individual; but when the process is prolonged and dysregulated, it leads to dysplastic changes.

Though the Schistosoma egg is a powerful inducer of cytokines, the exact mechanism of bladder carcinogenesis has yet to be elucidated. Several transcriptional factors including STAT 3 has been implicated in animal models linked to invasive bladder cancer [16, 18]. The bladder epithelium has been shown to have some protective agents such as bone morphogenic protein and Sonic hedgehog for regeneration following chemical or bacterial injury [16, 18, 19]. It is still unclear why schistosomiasis is associated more with squamous cell carcinoma than urothelial carcinoma. It is postulated that damage to the urothelium by *Schistosoma haematobium* ova leads to squamous metaplasia causing the strong association of squamous cell carcinoma.

Vitamin A (retinoic acid) deficiency has also been linked to Schistosoma-induced bladder cancer. Recent studies have demonstrated that retinoic acid receptor in the urothelium are essential in regeneration of the urothelium [20]. The fact that Schistosoma endemicity usually in areas of vitamin deficiency (including vitamin A) can postulate the vitamin A deficiency induced Schistosoma-associated bladder cancer in the sub-Saharan region.

## 5. Schistosomiasis, Coinfection, and Carcinogenesis

Helminthic infections in a host induces immunoregulatory cytokines (interleukin 10 (IL-10) and transforming growth factor-beta (TGF-B) which stimulate T helper cells 2 for immune response against helminth [16, 21]. This response may, however, lower the immunity against concurrent bacteria or virus infection, which can also stimulate carcinogenic changes. Viruses have been proven to produce oncogenes that alter the cell cycle, prevent apoptosis, and damage the DNA repair mechanism [16]. The human papillomavirus has been studied to play a role in Schistosoma bladder cancer. Uropathogens such as *Escherichia coli*, *Proteus mirabilis*, and *Klebsiella pneumoniae* associated with schistosomiasis may be procarcinogenic if high level of procarcinogenic N- nitrosamines is released in the urine [16]. A case-control study by Bedwani et al. [22] in Egypt was performed to assess the relation of urinary schistosomiasis, intestinal schistosomiasis, age, sex, occupational risk, smoking, and other urinary tract infection (UTI) to the formation of bladder cancer. Data from the study revealed that the clinical history of urinary schistosomiasis is significant but with modest risk of bladder cancer. This implies that other confounding risk factors are crucial to induce carcinogenesis in most cases of bladder.

## 6. Genetic and Immunohistochemical Profile of Schistosoma Bladder Cancer

Urothelial carcinoma is the most common histology of bladder cancer in the United States and Western Europe accounting for over 90% of cases. Most of the immunohistochemistry assays have been on urothelial carcinoma for this reason. Limited research on the molecular basis of Schistosoma bladder cancer has been published in the sub-Saharan region. However, in North Africa, especially Egypt, major strides have been made over the years to establish the molecular basis of schistosomiasis and bladder cancer. A case-control study by Hammam et al. [23] in Egypt assessing the expression of the fibroblast growth factor receptor (FGFR3) protein, gene amplification in bladder cancer, and associated schistosomiasis revealed that FGFR3 was considerably associated with bladder cancer tumor-grade and stage. Another data from Egypt by Elmansy et al. [24] assessing association of Fas and FasL in human bladder cancer with schistosomiasis found that the association of schistosomiasis with bladder cancer raised the incidence of Fas positive immunoreactivity to 100%. A report from Malaysia involving cohorts from the Middle East reviewed the role of genetic markers tumor protein (p53), p16, bcl-2, ki-67, c-myc, retinoblastoma tumor-suppressor gene (Rb), and epidermal growth factor receptor (EGFR), using the immunohistochemistry assay in Schistosoma bladder cancer (SBC) and nonschistoma bladder cancer (NSBC). The study revealed a distinct molecular profile and tumor progression for SBC and NSBC associated with these markers [25]. Another research by Warren and colleagues assessed mutations in the p53 gene in Schistosoma bladder cancer from 92 patients in Egypt where schistosomiasis is hyperendemic [26]. The results showed that there were mutations in exons 5 and 8 concluding that the excess of transitions along dinucleotides in Schistosoma bladder cancer resulted from nitric oxide produced by the inflammatory response induced by Schistosoma ova. These studies have tried to establish a molecular basis upon which a specific therapy for bladder cancer can be targeted.

## 7. Histopathological Evidence of Bladder Cancer and Schistosoma Ova in Sub-Saharan Africa

Current reports from Egypt reviewing 9,843 patients (1970–2007) found a decrease in the frequency of *S. haematobium* infection with concomitant decrease in squamous cell carcinoma. There was subsequent increase in urothelial cell carcinoma and an increase in the median age of patients diagnosed of bladder cancer [27]. This paradigm shift has been due to the introduction of a national schistosomiasis control program in Egypt. However, this risk is being replaced by increasing industrial chemical exposure and smoking causing the rise in urothelial cell carcinoma. In Egypt and other North African nations, SCC is more common in men (the male to female ratio ranges from 3 : 1 to 5 : 1), perhaps males performing agricultural work are more exposed to schistosomiasis-infested water [28]. In many sub-Saharan regions, SCC of the bladder is proportionately similar in males and females. This is possibly due to equal schistosomiasis exposure of girls and boys. Women from the sub-Saharan regions fetch household water and perform most agricultural tasks as well as their male counterparts. Although squamous cell carcinoma of the bladder frequently presents at a locally advanced stage, the tumors are generally well differentiated, with a rather low incidence of lymphatic and hematogenous metastases [28].

However, across sub-Saharan Africa, this shift may not be obvious for most settings. A one-year survey in Angola of 300 inhabitants to ascertain the prevalence of *S. haematobium* showed that the prevalence of *S. haematobium* in this population was 71.7% with most patients presenting with dysuria, hypogastric pain, and hematuria [27]. A review of 9 retrospective studies from Nigeria, Sudan, Zambia, Senegal, and Angola showed that squamous cell carcinoma of the bladder was the commonest histological type in most of these regions with an associated average of 40.5% histological evidence of Schistosoma ova [7–15]. Most of these reports were retrospective hospital-based studies and may not reflect the true epidemiological profile of Schistosoma bladder cancer in those regions entirely.

**Figure 1:**
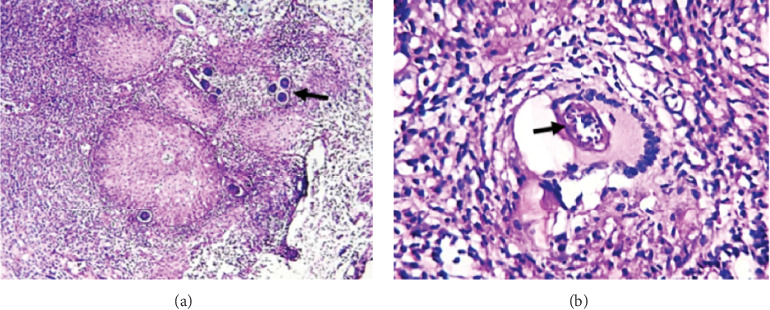
Histological slide of bladder Schistosoma infection (hematoxylin/eosin × 150). (a) The black arrow shows Schistosoma ova disseminated in the bladder with abundant leukocytic infiltrates. (b) Schistosoma granuloma with black arrows showing a Schistosoma ovum phagocytized by a giant cell.

A review of bladder tissue pathology at major referral center in Senegal from 2013 to 2018 revealed 150 patients with bladder cancer accounting for 96.7% of bladder specimen and 3.3% of all cancer within that period. The predominant histological type was urothelial cell carcinoma (87.7%) associated with 10% histological evidence of Schistosoma ova, while squamous cell carcinoma (9.2%) was associated with 42% histological evidence of Schistosoma ova (Figures 1(a)-1(b)). With rapid industrialization and smoking amongst adults in Senegal, it is possible that the histology could be more of urothelial cell carcinoma. Evidence have shown that an effective control of schistosomiasis, in settings where squamous cell bladder cancers are common, leads to changes of the histological types of cancers towards a western type (urothelial bladder cancer). However, an early data from 1950 to 2005 in Senegal of 428 patients assessing the epidemiological and histological profile of bladder cancer revealed that the leading histological type was SCC (50.7%), and 29.2% of cases had histological evidence of Schistosoma ova [13]. A retrospective review of 185 patients in Tanzania by Rambau et al. showed that among all bladder cancer detected, 44.9% had schistosomes eggs. The eggs were calcified with fibrosis around the eggs, signifying an old infection. Moreover, such eggs were more commonly found in histology of squamous cancers compared to nonsquamous cancers with a significant difference (*p* < 0.001). This explains that schistosomiasis may be responsible for a large burden of urinary bladder cancers in the region studied.

The review of bladder cancer pathology in SSA shows that SCC tends to be focally located as an ulcerative and nodular lesion along the bladder fundus. These masses are usually >3 cm in size at first presentation as reported in several series from SSA [5]. A 10-year retrospective analysis by Mohammed et al. in Nigeria showed that SCC is also muscle invasive in 80% of cases at the time of first presentation [11]. Contrarily, urothelial bladder cancer appears to be multifocal, small, and papillary with a lower frequency of muscle invasion at first presentation for about 70%–85% as shown by Forae et al. [29] and Biluts and Minas [30].

A review analysis by Bowa et al. (2018) has shown that the major histological type in Africa is SCC, and these tumors are locally invasive spreading from the fundus to the bladder neck rather than the transmural spread seen in urothelial bladder cancer [5]. Based on this concept, it is appropriate that these tumors are best managed with radical cystectomy than the conventional transurethral resection of bladder tumor (TURBT) for nonmuscle invasive urothelial bladder tumor. These patients are relatively younger and healthier, with minimum risk of urethral recurrence, unlike urothelial cancer [6].

Evidence from the National Cancer Registry in Egypt have shown that urothelial bladder tumor has a better 5-year survival of approximately 90% as compared to SCC, which has a 5-year survival close to 70% [31]. Up to 27% of SCC may be fixed and inoperable, especially if located below intraureteric bundle of Mercier [32].

## 8. Control of Schistosomiasis

The dismal control of primary exposures for bladder schistosomiasis is further perplexed by an absence of institutionalized population-based cancer screening programs. This contributes to a late presentation of cases, typically establishing a lower prospect of curative treatment. Several urology centers still lack basic endoscopic equipment. The lack of skilled personnel and training facilities to support “basic” procedures such as the transurethral resection of bladder tumor (TURBT) impedes the best standard of care [2].

There are ongoing research of schistosomiasis and bladder cancer, but there is still a gap of knowledge between this infection and its morbidity [26]. Currently with the introduction of praziquantel, there has been a modest fall in the incidence of the disease. However, most nations that achieved eradication of schistosomiasis did not use chemoprophylaxis alone but rather associated significant socioeconomic, public health, and environmental changes [26].

The prevalence of *Schistosoma haematobium* infection after 14 months of treatment and chemoprophylaxis fell from 58.9% to 5.8%, and the rate of heavy infection significantly reduced from 40.0% to 18.9% after intervention in Yemen [33]. A national control program for neglected tropical disease including schistosomiasis was introduced in Uganda during March 2003 [34]. Annual chemoprophylaxis for Schistosoma infection was provided to school children in endemic region and adults in selected communities where local prevalence of Schistosoma in school children was high [34]. The result of treatment showed a significant reduction in the prevalence of Schistosoma infection in school children across 3 regions in the country. *Schistosoma haematobium* is still endemic in about 53 countries in the Middle East and most part of the African continent, including the islands of Madagascar and Mauritius. Due to the successful eradication programs, the infection is no more of significant public health concern in Egypt, Lebanon, Oman, Syria, Tunisia, and Turkey because of low or nonexistent transmission rate [34].

With many financial and economic challenges in the Saharan region, focus should be placed on reducing the morbidity of Schistosoma before long-term eradication. Effective public health awareness, environmental sanitation, behavioral changes, and chemoprophylaxis are parameters to explore.

## 9. Conclusion

There is some evidence to support the association between schistosomiasis and bladder cancer. The magnitude of the evidence is, however, limited by the existent literature composed mainly of retrospective data. Bladder cancer pathology has evolved with all the efforts to reduce the incidence of schistosomiasis and the prominence of other risk factors. There is a need for more genomic and molecular research to better characterize *S. haematobium* and its effects on the bladder. Such goal will contribute immensely to Schistosoma bladder cancer prevention and control.

## Figures and Tables

**Table 1:** Histopathological review of bladder cancer and Schistosoma ova in sub-Saharan Africa.

Study	Study method and objectives	Findings and conclusion
Botelho et al. 2015 (Angola) [7]	One-year survey in Angola of 300 inhabitants to ascertain the prevalence of *S. haematobium*	The prevalence of *S. haematobium* in this population was 71.7% with predominantly dysuria, hypogastric pain, and hematuria

Husain et al. 2008 (Sudan) [8]	One-year retrospective study evaluating the risk factors of bladder tumor in Sudan	84.6% of patients with SCC had a urinary schistosomiasis with Schistosoma egg seen on histology

Mapulanga et al. 2013 (Zambia) [9]	A prospective cross-sectional study assessing the epidemiology, associated infection, and histology of bladder cancer	60.4% of the histology was SCC, and of these, 43.8% has associated Schistosoma infection

Mungadi et al. 2007 (Nigeria) [10]	5-year retrospective review of the epidemiology of bladder cancer in Northwestern Nigeria	SCC accounted for 65.1% of all bladder tumors, and 50% of these cases had histological evidence of chronic urinary schistosomiasis

Mohammed et al. 2012 (Nigeria) [11]	5-year histopathological review of Schistosoma infection	Urinary bladder schistosomiasis was the commonest site 62.6% with 30% associated bladder cancer

Rambau et al. 2013 (Tanzania) [12]	10-year retrospective study to assess the burden of schistosomiasis and bladder cancer	The leading histological type was SCC (55.1%), and 73.5% of cases were associated with schistosomiasis

Diao et al. 2008 (Senegal) [13]	Retrospective study of 428 patients assessing the epidemiological and histological profile of bladder cancer in Senegal	The leading histological type was SCC (50.7%), and 29.2% of cases had histological evidence of Schistosoma ova

Ibrahim et al. 2015 (Nigeria) [14]	Retrospective study of 144 patients reviewing the clinical and histological pattern of bladder cancer	The commonest histological type was SCC (63.9%), and 41.7% of cases had histological evidence of Schistosoma ova

Ochicha et al. 2003 (Nigeria) [15]	4-year retrospective study of 89 patients assessing the histological profile of bladder cancer	The commonest histological type was SCC (53.0%), and 21.3% of cases had histological evidence of Schistosoma ova
